# Autophagic Regulation of Lipid Homeostasis in Cardiometabolic Syndrome

**DOI:** 10.3389/fcvm.2018.00038

**Published:** 2018-05-03

**Authors:** Mingjie Yang, Yingmei Zhang, Jun Ren

**Affiliations:** ^1^Department of Cardiology and Shanghai Institute of Cardiovascular Diseases, Zhongshan Hospital, Fudan University, Shanghai, China; ^2^Center for Cardiovascular Research and Alternative Medicine, University of Wyoming College of Health Sciences, Laramie, WY, United States

**Keywords:** heart, autophagy, lipid metabolism, lipophagy, cardiometabolic diseases

## Abstract

As an important protein quality control process, autophagy is essential for the degradation and removal of long-lived or injured cellular components and organelles. Autophagy is known to participate in a number of pathophysiological processes including cardiometabolic syndrome. Recent findings have shown compelling evidence for the intricate interplay between autophagy and lipid metabolism. Autophagy serves as a major regulator of lipid homeostasis while lipid can also influence autophagosome formation and autophagic signaling. Lipophagy is a unique form of selective autophagy and functions as a fundamental mechanism for clearance of lipid excess in atherosclerotic plaques. Ample of evidence has denoted a novel therapeutic potential for autophagy in deranged lipid metabolism and management of cardiometabolic diseases such as atherosclerosis and diabetic cardiomyopathy. Here we will review the interplays between cardiac autophagy and lipid metabolism in an effort to seek new therapeutic options for cardiometabolic diseases.

## Cardiometabolic Syndrome

Cardiometabolic syndrome, also termed metabolic syndrome (MetS), is a clinical group of inter-related risk factors associated with atherosclerotic cardiovascular disease (ASCVD) and other metabolic diseases such as type 2 diabetes mellitus and stroke. The major risk factors for MetS include abdominal obesity, dyslipidemia, hypertension, insulin resistance and glucose intolerance (1). According to the National Health and Nutrition Examination Survey (NHANES), the age-adjusted prevalence of cardiometabolic syndrome was 22.9% (95% CI: 20.3 to 25.5%) between 2009 and 2010 in adults (≥20 years old) in the United States, with a prevalence of hypertriglyceridemia, elevated blood pressure, hyperglycemia and elevated waist circumference at 24.3, 24.0, 19.9 and 56.1%, respectively (2). Likewise, data from the International Diabetes Federation (IDF) supported that approximately 25% of adults worldwide suffer from metabolic syndrome, especially in those with obesity (with a rate of 60%) (3).

Despite of the improved understanding of the risk factors, the underlying mechanism(s) of cardiometabolic syndrome remains elusive at this point. A number of possible theories have been postulated including genetic and epigenetic factors, oxidative stress, apoptosis, insulin resistance, endothelial dysfunction and dysregulated lipid metabolism (4, 5). Recent findings have also suggested a key role of dysregulated autophagy in the pathophysiological change seen in cardiometabolic syndrome. Nonetheless, it remains unknown whether defective autophagy is a cause or result of cardiometabolic syndrome (6). As dyslipidemia acts as a key component of cardiometabolic syndrome, we will briefly discuss the complex interplay between autophagy and lipid metabolism, with an emphasis on cardiovascular diseases among various complications of cardiometabolic syndrome.

## Autophagy and Its Dysregulation

**Figure 1:**
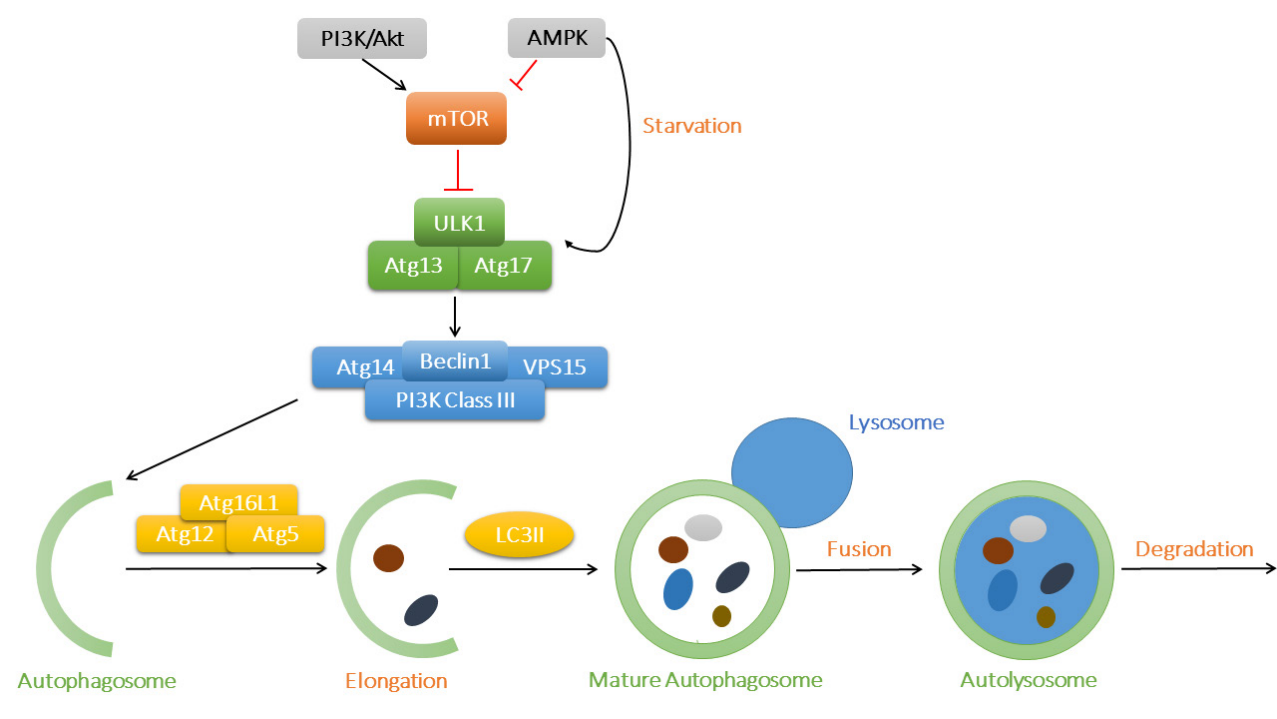
The process of macroautophagy and its main signaling regulatory mechanisms. The key regulator mTOR binds to ULK1 to suppress its activation, while AMPK and PI3K/Akt act as the primary upstream signaling regulatory molecules for mTOR. AMPK might be activated in starvation to promote the initiation of autophagy. ULK1 complex then activates Beclin1 complex and contributes to autophagosome nucleation. Atg12, Atg5 and Atg16L1 complex is involved in the elongation of autophagosome membrane, with LC3II being recruited into expanding cargos. Finally, mature autophagosomes fuse with lysosomes and generate autolysosomes, where waste protein aggregates and organelles were broken down into raw materials such as amino acids and lipids.

Autophagy is classified into three types, including macroautophagy, microautophagy and chaperone-mediated autophagy. In macroautophagy (or autophagy thereafter), autophagosomes transfer aged or damaged cellular cargo components and organelles to lysosomes for degradation, which serves as an important recycling process to maintain cellular homeostasis. As depicted in Figure 1, mammalian target of rapamycin (mTOR) is the key regulator in the autophagy pathway. With sufficient nutrient supply, mTOR binds with UNC-51-like kinase 1 (ULK1) and inhibits the initiation of autophagy. However, AMP-activated protein kinase (AMPK) is activated under starvation, which promotes autophagy through phosphorylation of ULK1. The ULK1 complex (ULK1-Atg13-Atg17) turns on the Beclin1 complex [Beclin1-Atg14-Vps34/Class III phosphoinositide 3-kinase (PI3K)-Vps15], fostering autophagosome nucleation. Atg12, Atg5 and Atg16L1 bind together with the help of Atg7 and Atg10, which promotes the elongation of autophagosome. LC3II (microtubule-associated protein 1 light chain 3) is also recruited into the growing membranes during this process. Finally, mature autophagosomes fuse with lysosomes and form autophagolysosomes, where damaged proteins and organelles are degraded and the breakdown products released into cytoplasm (7).

Apart from removal of unnecessary protein aggregates and organelles, degradation products such as amino acids and lipids also serve as materials for new synthesis in nutrient deprivation (8). Besides, autophagy also plays an essential regulatory role in lipid metabolism (or lipophagy), lipoprotein assembly and metabolic homeostasis (9). Abundant experiments and clinical evidence has shown that impaired autophagy disturbs cellular homeostasis and therefore contributes to the onset and development of many metabolic diseases including obesity, diabetes, atherosclerosis, and steatohepatitis (10, 11).

## Autophagy Regulates Lipid Metabolism

Lipid overload is a pivotal element of cardiometabolic syndrome and recent evidence has suggested a role for defective autophagy in the dysregulation of lipid metabolism (12). Various genetically engineered murine models of autophagy deficiency have been employed in an effort to unveil the precise role of autophagy in metabolic diseases. For example, deletion of p62 led to the increase of body fat and insulin resistance (13, 14). Fibronectin type III domain containing protein 5 (FNDC5) deficiency impaired autophagy and fatty acid oxidation, as well as enhanced lipogenesis through AMPK-mTOR pathway, the effect of which was rescued by rapamycin to restore autophagy (15).

**Figure 2:**
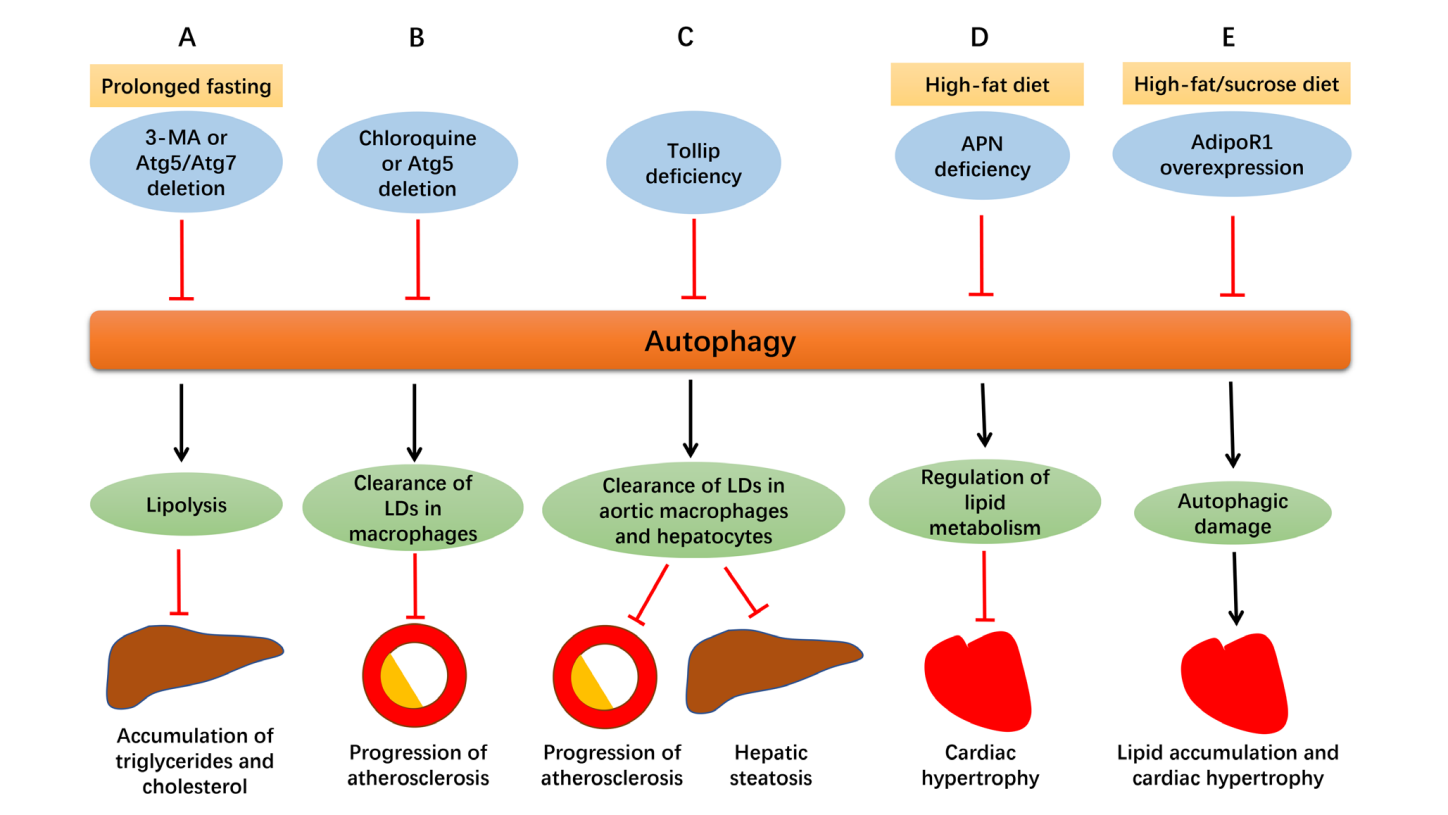
Defective autophagy alters lipid homeostasis. **(A)** In prolonged fasting, suppressed autophagy by 3-MA or genetically ablation of Atg5 or Atg7 promotes delivery of fatty acids to the liver with suppressed hepatic lipolysis at the same time, resulting in the accumulation of triglycerides and cholesterol in the liver. **(B)** Suppressed autophagy by chloroquine or Atg5 deletion in macrophages disrupts cholesterol efflux, leading to excessive lipid droplets (LDs) deposition and ultimately, enlargement of atherosclerotic plaques. **(C)** Tollip deficiency impairs lipophagy and contributes to lipid accumulation in aortic macrophages and hepatocytes, which aggravates atherosclerosis and hepatic steatosis. **(D)** Adiponectin (APN) deficiency disturbs autophagy and leads to accentuation of obesity and cardiac hypertrophy in high-fat diet intake. **(E)** In the face of prolonged high-fat/sucrose diet intake, AdipoR1 overexpression disrupts excessive autophagy, alleviates lipid accumulation and lipotoxicity, and retards cardiac hypertrophy.

Autophagy influences lipid metabolism in a number of ways, from lipogenesis to lipolysis (16). Autophagy and lipolysis are both decreased with enough food supply, while both are increased in nutrient deprivation (17). As depicted in Figure 2, in prolonged fasting, using 3-methyladenine (3-MA) or genetically ablation of Atg5 or Atg7 increased fatty acid transfer to the liver, along with suppressed hepatic lipolysis, thus leading to the accumulation of triglycerides and cholesterol in the liver (18, 19). Besides, inhibition of Notch signaling using DAPT triggered early autophagy via phosphatase and tensin homolog (PTEN)-PI3K/AKT/mTOR pathway and led to adipogenic differentiation from human bone marrow mesenchymal stromal cells (MSCs), the effect of which was abolished by chloroquine or 3-MA (20). It was also reported that chronic stress prevented MSCs from differentiating into adipocytes due to the inhibited autophagy and elevated CD99 (21).

Apart from lipid metabolism, autophagy also plays an essential role in the regulation of lipoprotein metabolism. Lipoprotein contains hydrophobic lipids and amphipathic proteins in order to transfer lipids in circulation, among which low-density lipoprotein (LDL) is commonly considered an independent risk factor of cardiovascular diseases while high-density lipoprotein (HDL) is deemed cardioprotective (17). It was found that phospholipid sphingosine-1-phosphate (S1P) might be a novel mechanism of HDL cardioprotection. HDL-S1P binds with S1P receptors in heart and activates PI3K/AKT signaling pathway, which would suppress autophagic damage and improve heart function in pressure-overload heart failure (22, 23). What’s more, mechanical stretch induced cardiac hypertrophy and elevated autophagy as manifested by LC3II and Beclin1, through upregulating angiotensin II receptor 1 (AT1) receptor. HDL inhibited mechanical stress-induced autophagy and mitigated cardiac hypertrophy via the AT1-PI3K/AKT pathway (24). Besides, apoB100 is a protein involved in VLDL (low-density lipoprotein) and LDL formation. Disruption of apoB100 degradative pathway results in the increase of plasma triglycerides and LDL. Omega-3 poly-saturated fatty acids promote autophagic degradation of apoB100 and suppress VLDL accumulation, denoting a potential therapeutic target for lipid disorders (25). In conclusion, autophagy may play an indispensable role in regulating lipid metabolism, including lipogenesis, lipolysis and adipogenic differentiation from multipotential stem cells. Besides, autophagy is also involved in governing lipoprotein metabolism. As a good example, lowered autophagic damage may contribute to the cardioprotective role of HDL.

## Autophagy Induced by Lipids

Autophagy may be stimulated by saturated or unsaturated fatty acids such as palmitate and oleate. Levels of Beclin1 and Atg7 were elevated in adipose tissues following a prolonged high-monounsaturated fatty acid diet intake (26). Palmitate induced autophagy in various cell types and EIF2AK2/PKR (eukaryotic translation initiation factor 2α kinase 2/protein kinase R) and STAT3 (signal transducer and activator of transcription 3) may be involved in the facilitated autophagy process. Genetic or pharmacological inhibition of STAT3 stimulated autophagy both *in vitro* and *in vivo*, while overexpression of STAT3 inhibited starvation-induced autophagy, possibly through its interaction with the dsRNA-activated protein kinase (PKR). STAT3 acted as a competitive inhibitor of PKR to inhibit PKR phosphorylation. Palmitate is capable of disrupting the inhibitory STAT3-PKR interactions and led to phosphorylation of PKR-dependent EIF2α, which promoted autophagic induction (27, 28).

Diabetic cardiomyopathy is a common complication in type 2 diabetes mellitus patients, featured by cardiac hypertrophy and heart failure. Russo and colleagues used milk fat-based diet consisting of abundant saturated fatty acids (SFA) to induce diabetic cardiomyopathy-like hypertrophy and left ventricular dysfunction in mice. SFA diet promoted autophagy indicated by increased LC3II and Beclin1, and sphingolipids are required in the pathogenesis (29). Moreover, dietary lipids are packaged and stored mainly in triglyceride-containing droplets in enterocytes, which triggers autophagy instantly for lysosomal degradation (30). Likewise, autophagy can be stimulated by oxidized lipids such as 4-hydroxynonenal (4-HNE) and oxidized (ox) LDL in advanced atherosclerotic plaques (31). These findings suggest that autophagy can be induced by fatty acids and lipids through various mechanisms.

## Lipophagy and Drug Targets for Atherosclerosis

Intracellular lipids including triglycerides and cholesterol are stored in the form of lipid droplets (LDs). Lipids may induce autophagy and undergo autophagic-lysosomal degradation in order to avoid lipotoxicity caused by excessive lipid accumulation, which is often termed as lipophagy, a special form of selective macroautophagy. LDs are transferred by autophagosomes to lysosomes and degraded into free fatty acids and cholesterol, and defective lipophagy results in excessive lipid accumulation. Besides, the amount of lipids targeted for lipophagy varies according to different nutritional status. Studies showed increased association of LC3 with LDs under starvation, and the percentage of autophagosomes containing lipids increased markedly with increased time of energy deprivation. Lipophagy is selectively upregulated when facing extra energy needs, while the breakdown products free fatty acids undergo β-oxidation to supply ATP. Increased lipophagy enables the cell to generate energy timely for cell survival under starvation (32). Therefore, lipophagy regulates not only intracellular lipid stores, but also energy homeostasis especially in face of nutrient deprivation.

As commonly known, accumulation of lipids and lipoproteins are important early pathophysiological changes in atherosclerosis, and macrophage is closely involved in the disease. Atherosclerosis usually begins with the retention of the lipoproteins into the subendothelial space, where they are oxidized and accumulate, leading to the formation of plaques gradually. For example, apoliprotein B enters subendothelial space of the artery wall and triggers secretion of inflammatory cytokines, and then monocytes are attracted here and differentiate into macrophages. Accumulated lipids and lipoproteins are engulfed by macrophages to form foam cells, which contributes to progression of atherosclerotic plaques (33). Autophagy serves as a significant mechanism for clearance of lipid excess in these plaques. Lipids are carried to lysosomes by autophagosomes where they are degraded into free cholesterol and released out of macrophages (34). As depicted in Figure2, impaired autophagy either by chloroquine or Atg5 deletion in macrophages disrupted cholesterol efflux to apolipoprotein A-I (ApoA-I) and led to accumulation of intracellular lipid droplets and macrophage dysfunction, which resulted in progression of atherosclerosis eventually. Macrophage lipophagy and cholesterol efflux was upregulated both *in vitro* and *in vivo* in response to lipid excess, which may become a novel therapeutic target for atherosclerosis (35).

As mentioned above, impaired lipophagy leads to intracellular lipid accumulation, thus contributing to atherosclerosis and hepatic steatosis (36). As shown in Figure2, toll-interacting protein (Tollip), a molecule associated with autolysosome fusion, is believed to play an essential role in this pathological process. Deletion of both ApoE and Tollip disturbed the fusion of lipid droplets with lysosomes in aortic macrophages and hepatocytes, and aggravated atherosclerosis and hepatic steatosis, compared to deletion of ApoE alone. It may be concluded that Tollip deficiency may impair lipophagy, and contribute to lipid accumulation and enlargement of atherosclerotic plaques (36). Another report suggested that inhibition of mTOR offers anti-atherosclerotic property through activation of autophagy and cholesterol efflux and depletion of macrophages in plaques. However, lipid stores are reduced with increased LDL levels at the same time, which may become a side effect if utilized as an anti-atherosclerotic therapy (37).

Moreover, ORMDL sphingolipid biosynthesis regulator 3 (ORMDL3), as an essential regulator of lipid metabolism, inflammation and ER stress, is involved in the pathogenesis of atherosclerosis. Expression levels of ORMDL3 were elevated in the Chinese Han population carrying alleles of the rs7216389 and rs9303277, exhibiting overtly elevated atherosclerotic risk. Experimentally, oxidized low-density lipoprotein (ox-LDL) stimulated ORMDL3 expression in endothelial cells. ORMDL3 silencing reduced basal and ox-LDL-induced autophagy, and suppressed BECN1 expression, which is a protein vital to autophagic initiation. Therefore, ORMDL3 mediates ox-LDL-induced autophagy in endothelial cells in atherosclerosis (38).

Based on these findings, it is concluded that lipophagy serves as a significant mechanism for the clearance of excessive lipids, maintenance of cellular homeostasis, and prevention against the progression of atherosclerotic plaques. Impaired lipophagy causes accumulation of lipids and lipoproteins, thus autophagy may be considered a possible therapeutic target for atherosclerosis.

## Mitophagy and Dyslipidemia

Mitochondria are organelles responsible for energy supply and are pivotal to cell survival, in particular in organs with a great need for energy such as the heart. Mitochondrial dysfunction causes profound damage to cellular homeostasis, necessitating the need for mitochondrial quality control. Mitophagy serves as an indispensable mechanism to transfer damaged mitochondria for lysosomal degradation by autophagosomes in order to clear aberrant mitochondria in metabolic diseases. Regulatory machineries of mitophagy are involved with PTEN-induced putative kinase 1 (PINK1), Parkin (the E3 ligase Parkinson protein-2), Mfn2 (mitochondrial fusion 2 protein mitofusin), the Nix/Bnip3L-Atg8-LC3II complexes and Fun14 domain containing 1 (FundC1) (39). PINK1 phosphorylates Mfn2, accumulates on damaged mitochondria and recruits Parkin to mitochondrial outer membrane, which initiates Mfn2 degradation and mediates the clearance of defective mitochondria ultimately (40).

As mitophagy plays a vital role in the clearance of unwanted mitochondria, impaired mitophagy was closely associated with mitochondrial injury and dyslipidemia in cardiometabolic diseases such as atherosclerosis (41, 42). Inflammation is widely accepted to be a key factor in the formation of atherosclerotic plaques and defective mitophagy activated inflammation, which led to secretion of inflammatory cytokines such as IL-1β. One possible mechanism may be due to the inability of defective mitophagy to clear damaged mitochondria, resulting in production of superoxide/ROS (reactive oxygen species), inflammation and disrupted lipid metabolism, leading to the ultimate plaque expansion. Though limited reports are available at this time, it is plausible to credit the essential roles for mitophagy in the regulation of lipid metabolism and thus outcome of cardiometabolic diseases.

## Conclusion and Discussion

Autophagic-lysosomal degradation pathway is an indispensable mechanism for clearing and recycling waste cellular components to maintain homeostasis and to provide materials for new synthesis. Recent evidence has emphasized a vital role for autophagy in lipid metabolism in cardiometabolic diseases such as atherosclerosis. Lipophagy, as a selective form of autophagy, takes charge of translocating lipids for lysosomal degradation, and prevents excess lipid deposit in macrophages and expansion of atherosclerotic plaques, which should be a promising drug target for the management of cardiometabolic diseases.

It is noteworthy that the role of autophagy in cardiometabolic diseases can be complex. Different reports indicate both protective and detrimental roles in atherosclerosis. Most studies do favor a protective role for autophagy in the prevention of atherosclerosis (31, 43). Autophagy of SMCs (smooth muscle cells) in fibrous caps of advanced atherosclerotic lesions helps to degrade damaged components caused by oxidative stress or other injury, thus maintaining plaque stability (44). Still, cellular damage will accumulate if too much or persistent oxidative stress exists. The damaged lysosomal membranes are unable to fuse with autophagosomes and autophagy no longer works. However, autophagic death of SMCs and endothelial cells will result in plaque destabilization and thrombosis and deteriorate the disease, which means excessive autophagy can also be detrimental during this pathophysiological change (45).

Apart from atherosclerosis, autophagy also plays both beneficial and detrimental roles in other cardiometabolic diseases. As depicted in Figure 2, adipocyte-derived cytokine adiponectin (APN) and its receptor 1 (AdipoR1) are essential for the regulation of lipid metabolism in cardiometabolic diseases through altering autophagic process. APN is cardioprotective in high-fat diet induced obesity and APN deficiency impaired autophagy, which caused accentuation of obesity, metabolic intolerance, cardiac hypertrophy and dysfunction (46). In another study with 6 month high-fat/sucrose diet (HFSD) treatment, autophagic genes Beclin1 and Lamp2A were upregulated in cardiomyocytes, which was believed to be detrimental to heart since prolonged HFSD feeding led to lipotoxicity and cardiomyopathy. AdipoR1 overexpression disrupted excessive autophagy, reduced lipid accumulation and cardiac hypertrophy, and ameliorated cardiac function, which may suggest that its cardioprotective role is attributed to decreased autophagic damage (47).

In summary, basal level of autophagy is of great significance to clear damaged cellular components and maintain lipid homeostasis, while excessive autophagy may be detrimental and leads to cell death. Due to its regulatory effects on lipid metabolism, autophagy and lipophagy is considered a novel therapeutic target for cardiometabolic syndrome and atherosclerosis, which deserves to be explored further more.

## Author Contributions

MY drafted and revised the manuscript. YZ revised the manuscript. JR revised the manuscript and provided financial support.

## Conflict of Interest Statement

The authors declare that the research was conducted in the absence of any commercial or financial relationships that could be construed as a potential conflict of interest.
